# Status and associations of transition shock among nursing students during clinical practice: A cross-sectional study

**DOI:** 10.1371/journal.pone.0313524

**Published:** 2025-02-04

**Authors:** Yinying Tang, Xiuwen Chen, Yuying Liao, Tingyu Zheng, Yao Xiao, Yunhui You

**Affiliations:** 1 Teaching and Research Section of Clinical Nursing, Xiangya Hospital, Central South University, Changsha, China; 2 National Clinical Research Center for Geriatric Disorders, Xiangya Hospital, Central South University, Changsha, China; 3 Xiangya Nursing School, Central South University, Changsha, China; 4 School of Nursing, Southern Medical University, Guangzhou, China; 5 Department of General Surgery, Xiangya Hospital, Central South University, Changsha, China; 6 Department of Rheumatology and Immunology, Xiangya Hospital, Central South University, Changsha, China; KSAU-HS: King Saud bin Abdulaziz University for Health Sciences, EGYPT

## Abstract

**Aim:**

This study aimed to investigate the current state and influencing factors of transition shock among nursing students during clinical practice.

**Background:**

Transition shock among nursing students can significantly impact their academic performance and well-being. Understanding the key factors contributing to this shock is crucial for developing effective support strategies and improving overall educational outcomes.

**Methods:**

This cross-sectional study was conducted on October 8–28, 2022 at four tertiary Class A hospitals in Changsha, Hunan Province, located in south-central China. A convenience sample of 620 full-time nursing students was surveyed to collect demographic information and assess their transition shock levels using the transition shock scale. Data analysis included descriptive statistics, nonparametric tests, correlation analysis, and multiple regression. STROBE checklist was used for the methodology in this study.

**Results:**

A total of 564 nursing students were ultimately included in the study. The average overall transition shock score was 46 (41, 52). Attitude toward the nursing profession had an independent influence on nursing students’ transition shock (*p* < 0.05). Additionally, the number of night shifts, choosing nursing as the first choice, being class leaders, education level, future plans, school scale, and monthly household income contributed to different dimensions of transition shock (*p* < 0.05).

**Conclusion and implications for nursing policy:**

Nursing students experience moderate transition shock, with attitude towards nursing as a key influencing factor. Clinical managers should implement targeted measures to better support nursing students improve their attitudes.

## 1. Introduction

Nursing is an applied discipline based on practice [1]. Nursing clinical practice is an important part of practical teaching and a necessary path for nursing students to transform into nurses. It is also a transition from theory to practice for nursing students that directly affects whether they can work independently as soon as possible in the future [2]. During clinical practice, nursing students are faced with dual changes in their role and environment [3]. Due to the unfamiliar hospital environment, lack of clinical experience, and lack of mature knowledge and skills, they are prone to become nervous, diffident, self-doubting, and disoriented. Problems such as nurse‒patient conflicts may even occur. Nursing students have difficulty adapting to the identity of clinical caregivers and experience transition shock.

Transition shock refers to feelings of anxiety, instability, and insufficiency experience in the roles, responsibilities, relationships, knowledge, and expectations when moving to a new environment [4,5]. Clinical practice is a critical period for nursing students to fit into their roles. Some unadjusted students choose to quit the nursing profession after the practicum [6]. The shortage of nursing talent resources is a huge challenge faced by the global medical health system. A study in the United States found that as many as 35% of newly graduated nurses had intentions to leave their jobs due to poor transition [7], and in China, this proportion ranged from 20.2% to 56.1% [8]. Effective evaluation of the transition shock of nursing students is a prerequisite for improving their clinical practice experience and facilitating their smooth transition [9,10].

According to international and domestic research, the transition shock of nursing students is at a medium level [11,12]. One of the most influential factors of transition shock in nursing students is ambiguity in professional nursing values [13]. However, it is noteworthy that most existing literature on transition shock focuses on factors influencing newly graduated nurses. For example, a systematic review by Missen et al. [14] indicated that workload, work environment, support systems, and education and training are key factors affecting transition shock among new nurses. Notably, large-scale studies specifically addressing transition shock among nursing students, with detailed analyses of influencing factors, are scarce. To address this gap, the study aims to contribute to the existing body of knowledge by examining the current state and influencing factors of transition shock among nursing students using validated scales. However, in 2009, Duchscher proposed the Transition Shock Theory, which posits that when individuals transition from a student role to a nurse role, they experience feelings of confusion, doubt, and uncertainty in terms of physical, psychological, knowledge and skill, sociocultural, and developmental aspects. These challenges are influenced by their own knowledge, responsibilities, roles, and interpersonal relationships. The theory highlights that the clinical internship period is a crucial phase in the transition from nursing student to professional nurse and represents an optimal time for early assessment and intervention.

Regarding the measurement of transition shock, Kramer et al. [15] developed the environmental reality shock and related issues and concerns scale to measure the number of transition problems faced by newly graduated nurses and the degree of attention to these problems. Xue et al. [16] developed the transition shock of newly graduated nurses scale to study nurses’ level of transition shock. The Korean scholars Kim et al. [17] created a transition shock scale for undergraduate nursing students in 2019. Huang et al. [18] translated this scale into Chinese and tested its reliability and validity, demonstrating good psychometric properties.

In summary, understanding the factors influencing transition shock and addressing them effectively is crucial for supporting nursing students during their clinical internships. Duchscher’s Transition Shock Theory underscores the complexity of this transition and highlights the internship period as a key stage for assessment and intervention. By investigating the current state and influencing factors of transition shock using validated scales, this study aims to provide insights and recommendations to mitigate and prevent transition shock, ultimately enhancing the transition experience for nursing students.

## 2. Methods

The recruitment period for this study is October 8–28, 2022. A cross-sectional study was conducted at four tertiary Class A hospitals in Changsha, Hunan Province, located in south-central China.

### 2.1. Design & setting

We applied a cross-sectional survey study design to investigate nursing students’ transition shock during internships in four Chinese tertiary Class A hospitals. These hospitals are among the most prestigious medical institutions in the region, known for their advanced medical services, extensive training programs, and high patient volumes. Their selection is particularly pertinent to this study as they represent high-quality clinical environments where nursing students experience a wide range of complex and challenging situations. By focusing on these leading hospitals, this study aimed to provide a comprehensive understanding of transition shock and adaptation processes faced by new nursing graduates in demanding and resource-rich settings. In addition, it is worth noting that in China, nursing higher education is provided at two levels: nursing education at the junior college level and at the bachelor level. In general, junior college nursing students learn clinical knowledge and skills for three years, while undergraduate nursing students require four years. All educational programs include internships for at least eight months of clinical practice and give access to the same nursing certificate. However, junior college nursing education focuses on cultivating students’ clinical practice skills, while undergraduate education aims to develop students’ clinical critical thinking and cultivate applied talent.

### 2.2. Participants & recruiting

Interns were eligible for participation if they met the following criteria: ① Aged ≥18 years; ② enrolled in nursing education;③ engaged in an internship. Participation was not part of the formal clinical placement assignments and was therefore voluntary [19]. The management of the nursing schools and hospitals was blinded with regard to whether students participated.

We provided instructions containing all the information necessary to make a decision about participating in this study (*i*.*e*., the introduction and objective of the study, the required time to complete the questionnaire, confidentiality, expectations, and rights). After reviewing the information, interested individuals provided written informed consent before participating. To maximize participation, follow-up communications were conducted to address any additional questions. The online questionnaire link was distributed to nursing students during their internship in four tertiary Class A hospitals by nursing staff who were unfamiliar with the research using their usual communication medium (WeChat or another online platform). This approach leveraged the convenience and accessibility of online data collection, which not only facilitated a broader reach but also allowed for real-time data gathering and reduced the potential for response bias.

### 2.3. Survey

The complete questionnaire comprised two sections: demographics and the transition shock scale. Demographic data were collected in a manner designed by the researchers and included gender, age, educational background, family status, whether the respondent was an only child, the scale of the respondent’s school, the grade of the hospital where the respondent practiced, the respondent’s first choice of major for the college entrance examination, clinical practice, nursing attitude, and development intention after graduation. The demographic section consisted of multiple-choice questions and fill-in-the-blank questions, with uniform instructions to explain the concepts, meanings, and methods of providing demographic variables. For example, The "school scale" referred to a key university, first batch of university, second batch of university and junior college as assessed by the Ministry of Education; and the grades of the practicing hospitals were grade III, grade II, grade I and ungraded as assessed by the Ministry of Health.

The transition shock scale was used to measure reality shock for nursing students and was modified and validated by Kim and Shin. The original scale was developed by Kim et al. to measure newly graduated nurses’ transition shock. After sinicization, it consisted of 18 entries across six dimensions: conflict between theory and practice (3 entries), overwhelming practicum workload (3 entries), loss of social support (2 entries), shrinking interpersonal relationships (3 entries), ambiguity in professional nursing values (5 entries), and incongruity between clinical practicum and personal life (2 entries). Each item was rated on a 4-point Likert scale (1 = “strongly disagree”; 2 = “disagree”; 3 = “agree”; 4 = “strongly agree”). The total score ranged from 18 to 72, with a higher score indicating greater transition shock during clinical practice. Regarding this scale, the content validity indices (CVI) of all items were > 0.78, and the scale-level content validity indices (S-CVI) of 0.987 were the acceptable standard. The reliability coefficient (Cronbach’s alpha) of the scale was 0.912, and the range of every dimension’s reliability coefficient (Cronbach’s alpha) was 0.657–0.887. The computed Cronbach’s alpha based on the data collected from our study was 0.919, indicating acceptable reliability.

### 2.4. Data collection

Data were collected using an online survey platform (Wenjuanxing), which guaranteed adherence to all regulations and legislation regarding data protection.

Convenience sampling was used to select nursing students during their internships from four tertiary Class A hospitals in Changsha as the research object. The inclusion criteria were as follows: 1) full-time nursing intern; 2) more than or equal to 2 weekends of clinical practice; 3) voluntary consent from the participant; and 4) general cognitive and understanding ability. The exclusion criteria were nonclinical nursing care providers due to illness or other matters. Considering that the regression analysis method was selected for this study, the sample size was recommended to be at least 10 times the number of explanatory variables [20].

To ensure the reliability and validity of the data, we implemented several quality control measures. Each participant could submit the survey only once per account. We also set a minimum completion time of two minutes based on preliminary testing; responses completed in less than this time were flagged and excluded. Additionally, the platform was secured with password protection, and attention-check questions were included to identify and remove careless responses. A total of 620 questionnaires were sent, and 56 were eliminated due to irrational answers or unreasonable internet protocol addresses. Ultimately, 564 valid questionnaires were returned, for a response rate of 90.3%. The recycling of questionnaire data was applied to test the structural validity, item differentiation, and internal consistency of the scale.

### 2.5. Data analysis

The data were analyzed using SPSS Statistics 26.0 (IBM Corp., Armonk, NY, USA). The normal distribution of the study variables was confirmed using the Kolmogorov‒Smirnov test (*p* > 0.05). Frequencies and percentages were computed to describe the enumeration data, such as participants’ gender and family situation. The average, standard deviation (SD), median, lower quartile (P_25_), and upper quartile (P_75_) were used to describe the measurement data, such as the time of clinical practice and the total scores of the scale. The Mann‒Whitney *U* test and Kruskal‒Wallis *H* nonparametric analysis were applied to compare the score differences in the transition shock of nursing students with various sociodemographic characteristics. Spearman’s correlation analysis was used to examine the correlations among the study variables. In addition, multiple linear regression was used to analyze the influencing factors of the transition shock of nursing students.

### 2.6. Ethical considerations

Prior to data collection, the study protocol was approved by the relevant institutional review committee (Approval was granted by the Ethics Committee of Xiangya Hospital, Central South University with registry number 2022100820). Written informed consent was obtained from each individual participant prior to the survey. Participants were informed that participation was voluntary, that their anonymity was guaranteed at all times, and that the collected data would only be coded into numbers. Moreover, no incentives were offered to the participants. All methods were performed in accordance with the Declaration of Helsinki guidelines (World Medical Association, 2013).

## 3. Results

### 3.1. Sociodemographic characteristics of the sample

A total of 564 nursing students from four tertiary Class A hospitals in Changsha participated in this study. A total of 499 of them, accounting for 88.48%, were female, and 65 of them, accounting for 11.52%, were male. The age of the respondents ranged from 18 to 30 years, with a median of 21 years. According to the results of the nonparametric analysis, nursing students with different monthly household incomes, school scales, future plans, attitudes toward nursing, and first choice of major had different scores for transition shock. The difference was statistically significant (p < 0.05). Further details are provided in Table 1.

**Table 1:** Comparison of transition shock scores of nursing students with different sociodemographic characteristics (*N* = 564).

Variables	N (%)	Median (P_25_,P_75_)	*Z/H*	*p*
Gender			-0.065	0.948
Male	65 (11.5)	45.00 (40.00,53.00)		
Female	499 (88.5)	46.00 (41.00,52.00)		
Family Location			-0.688	0.491
City	224 (39.7)	46.00 (40.00,52.00)		
Village	340 (60.3)	46.00 (41.00,52.00)		
Whether You are the Only Child of Your Parents			-0.061	0.951
Yes	125 (22.2)	47.00 (41.00,51.00)		
No	439 (77.8)	46.00 (41.00,52.00)		
Monthly Household Income			7.915	0.048
< 2500	115 (20.4)	44.00 (41.00,52.00)		
2500–5000	263 (46.6)	46.00 (40.00,52.00)		
5001–7500	118 (20.9)	48.00 (43.00,53.00)		
> 7500	68 (12.1)	45.00 (40.00,52.00)		
Department			10.463	0.164
Internal Medicine	133 (23.6)	46.00 (40.00,53.00)		
Surgery	177 (31.4)	45.00 (41.00,50.00)		
Gynecology & Obstetrics	51 (8.9)	47.00 (40.00,53.00)		
Pediatrics	29 (5.1)	49.00 (44.00,54.00)		
Emergency & ICU	61 (10.8)	47.00 (42.00,53.00)		
Psychology	4 (0.7)	46.00 (44.00,48.00)		
Operating Room	34 (6.0)	46.00 (40.00,50.00)		
Others	75 (13.5)	44.00 (39.00,50.00)		
Education			4.262	0.119
Undergraduate	274 (48.6)	46.00 (41.00,52.00)		
Junior College	288 (51.1)	45.00 (40.00,52.00)		
Technical Secondary School	2 (0.3)	\		
School Scale			10.635	0.014
Key University	21 (3.7)	48.00 (45.00,54.00)		
First Batch of University	107 (19.0)	47.00 (43.00,53.00)		
Second Batch of University	160 (28.4)	45.00 (40.00,50.00)		
Junior College	276 (48.9)	45.00 (40.00,52.00)		
Student Leader Experience in Class			-1.501	0.133
Yes	396 (70.2)	45.00 (40.00,52.00)		
No	168 (29.8)	47.00 (42.00,52.00)		
Attitude toward Nursing			100.366	< 0.001
Strongly Like	59 (10.5)	38.00 (31.00,45.00)		
Like	184 (32.6)	43.00 (39.00,49.00)		
Average	285(50.5)	48.00 (43.00,53.00)		
Dislike	24 (4.3)	54.00 (48.00,58.00)		
Strongly Dislike	12 (2.1)	56.00 (44.00,61.00)		
Whether Nursing Profession is Your First Choice for College Entrance Examination			-5.287	< 0.001
Yes	324 (57.5)	44.00 (40.00,50.00)		
No	240 (42.5)	48.00 (43.00,54.00)		
Future Plan			32.969	< 0.001
Clinical Nursing	254 (45.0)	45.00 (40.00,50.00)		
Advanced Study	152 (27.0)	45.00 (41.00,49.00)		
Nursing Education	15 (2.7)	51.00 (43.00,59.00)		
Profession Change	33 (5.9)	52.00 (46.00,57.00)		
Have Not Decided	108 (9.1)	47.00 (41.00,54.00)		
Others	2 (0.3)	\		

*Z/H*: The Mann‒Whitney *U* test or Kruskal‒Wallis *H* nonparametric analysis; \: No median and quartile.

### 3.2. The total score of the transition shock scale and the score of each dimension

The total score of the transition shock scale of nursing students was 46 (41,52), and the specific scores of each dimension are detailed in Table 2.

**Table 2:** The total score of the transition shock scale and the score of each dimension (*N* = 564).

Dimensions	Score		Theoretical Maximum
	N	Median (P_25_,P_75_)	
Total Score	564	46 (41,52)	72
Conflict between Theory and Practice (1–3 entries)	564	8 (7,9)	12
Overwhelming Practicum Workload (4–6 entries)	564	9 (8,10)	12
Loss of Social Support (7–8 entries)	564	4 (4,6)	8
Shrinking Interpersonal Relationships (9–11 entries)	564	7 (6,9)	12
Ambiguity in Professional Nursing Values (12–16 entries)	564	13 (11,15)	20
Incongruity between Clinical Practicum and Personal Life (17–18 entries)	564	4 (4,6)	8

### 3.3. Correlation in every dimension of the transition shock scale with other variables

There was a positive correlation between nursing students’ attitudes toward nursing and the dimension of conflict between theory and practice (*r* = 0.120, *p* = 0.004). There were positive correlations between the number of night shifts and the dimension of overwhelming practicum workload (*r* = 0.128, *p* = 0.030), between the workload dimension and the first choice of major (*r* = 0.132, *p* = 0.002), and between attitude and the workload dimension (*r* = 0.197, *p* < 0.001) among the nursing students.

In addition, attitude toward nursing (*r* = 0.256, *p* = 0.02) and first choice (*r* = 0.113, *p* = 0.007) were positively correlated with the dimension of loss of social support. For the dimension of shrinking interpersonal relationships, nursing students’ attitudes toward nursing (*r* = 0.322, *p* < 0.001), future plans (*r* = 0.104, *p* = 0.014), and first choice (*r* = 0.191, p < 0.001) were positively correlated. There were negative correlations between nursing students’ educational background and the dimension of shrinking interpersonal relationships (*r* = -0.104, *p* = 0.013) and between the school scale and the relationship dimension (*r* = -0.140, *p* = 0.001).

Attitudes toward nursing (*r* = 0.487, *p* < 0.001), future plans (*r* = 0.127, *p* = 0.002), monthly household income (*r* = 0.111, *p* = 0.009), and first choice (*r* = 0.286, *p* < 0.001) were positively correlated with the dimension of ambiguity in professional nursing values among nursing students. In addition, student leader experience (*r* = 0.131, *p =* 0.002), attitude toward nursing (*r* = 0.385, *p* < 0.001), future plans (*r* = 0.127, *p =* 0.002), and first choice (*r* = 0.197, *p* < 0.001) were positively correlated with the dimension of incongruity between clinical practicums and personal life. Further details are provided in Table 3, and the assignment of enumeration data is shown in S1 Table.

**Table 3:** Correlation in every dimension of the transition shock scale with other variables (*N* = 564).

Variables	*r*	*p*
**Conflict between Theory and Practice**		
Attitude toward Nursing	0.120	0.004
**Overwhelming Practicum Workload**		
Night Shift	0.128	0.030
First Choice	0.132	0.002
Attitude toward Nursing	0.197	< 0.001
**Loss of Social Support**		
Attitude toward Nursing	0.256	0.020
First Choice	0.113	0.007
**Shrinking Interpersonal Relationships**		
Attitude toward Nursing	0.322	< 0.001
Future Plan	0.104	0.014
Education	-0.104	0.013
School Scale	-0.140	0.001
First Choice	0.191	< 0.001
**Ambiguity in Professional Nursing Values**		
Attitude toward Nursing	0.487	< 0.001
Future Plan	0.127	0.002
Monthly Household Income	0.111	0.009
First Choice	0.286	< 0.001
**Incongruity between Clinical Practicum and Personal Life**		
Student Leader Experience	0.131	0.002
Attitude toward Nursing	0.385	< 0.001
Future Plan	0.127	0.002
First Choice	0.197	< 0.001

### 3.4. The influencing factor analysis of transition shock in nursing students

Measurement data with statistical significance and enumeration data with set dummy variables were subjected to multiple linear regression analysis. It was clear that the influencing factor for transition shock in nursing students was their attitudes toward nursing. Further details are provided in Table 4, and the assignment of enumeration data is shown in S2 Table.

**Table 4:** Multiple linear regression analysis of transition shock in nursing students (*N* = 564).

Variables		Standardized Regression coefficient	Standard Error	*t*	*P*
Constant		45.029	6.264	7.188	< 0.001
Monthly Household Income	2500–5000	0.748	0.926	0.808	0.420
	5001–7500	1.763	1.080	1.632	0.103
	> 7500	0.615	1.273	0.483	0.629
School Scale	First Batch of University	0.251	1.983	0.126	0.899
	Second Batch of University	-1.563	1.950	-0.802	0.423
	Junior College	-0.199	1.898	-0.105	0.917
Attitude toward Nursing	Strongly Like	-9.820	1.217	-8.067	< 0.001
	Like	-3.732	0.807	-4.507	< 0.001
	Dislike	3.958	1.800	2.199	0.028
	Strongly Dislike	2.767	2.582	1.072	0.284
Whether Nursing Profession is the First Choice for College Entrance Examination		0.075	0.835	1.605	0.109
Future Plan	Clinical Nursing	2.677	5.895	0.454	0.650
	Advanced Study	2.097	5.913	0.355	0.723
	Nursing Education	4.675	6.247	0.748	0.455
	Profession Change	6.128	6.058	1.012	0.312
	Have Not Decided	3.780	5.916	0.639	0.523

## 4. Discussion

To the best of our knowledge, this study represents a multicenter, large-scale investigation into the current state and influencing factors of transition shock among nursing students during clinical practice, thereby contributing valuable insights to the existing body of knowledge on this topic. Our study reveals that nursing students faced a moderate level of transition shock, with their attitude towards the nursing profession emerging as a significant independent factor. Additionally, the number of night shifts, choosing nursing as the first choice, being class leaders, education level, future plans, school scale, and monthly household income contributed to different dimensions of transition shock.

### 4.1. Nursing students’ transition shock was moderate

This study is accompanied by a preprint resource [21]. The investigation revealed that within this survey, the overall score of nursing students’ transition shock was moderate 46 (41, 52), with a median entry score of 2.56 (2.28,2.89). The results were similar to those of Wang et al. [11] and lower than those of Kim et al. [22] from Korea. This may be related to the source of the study subjects, the way nurses are trained, the healthcare environment, and the underlying national context in different countries. Currently, increasing research emphasizes the significance of transition shock. Studies by Li et al. [23] have shown that transition shock has a significantly negative influence on the humanistic practice ability of newly graduated nurses. Nursing managers and educators can mitigate the adverse effects of transition shock by enhancing the benefits of organizational socialization programs, ultimately improving the humanistic practice abilities of newly graduated nurses. This underscores the necessity of ongoing efforts to enhance support systems and educational practices, thereby facilitating smoother transitions and better equipping students for their professional roles.

### 4.2. The influencing factors in each dimension of the transition shock scale were different

The transition shock scale contained six dimensions, and each dimension had a different degree of impact on nursing students. According to the results of the correlation analysis, the influencing factors of each dimension were different.

#### 4.2.1. Attitudes toward nursing were associated with the dimension of conflict between theory and practice

In a study by Kim et al. [22], nursing students experienced a difference between what they practiced in the clinical setting and the classroom. As the concept of clinical nursing continues to evolve, new clinical technologies and emerging businesses arise [4], frequently leading to conflicts between theory and practice in the workplace. In a study by Sin and Kim [13], new nurses encountered confusion and embarrassment as they found that the knowledge they learned at school differed from practice. In addition, senior nurses showed a lack of professionalism by not following principles when performing nursing, which is consistent with previous results [24]. The preceptors’ behaviors directly influenced nursing students’ practice so that nursing students, who already lacked practical experience and self-confidence, became negative about clinical work and less favorable to the nursing profession. On the one hand, preceptors should communicate more with nursing students to increase their confidence in clinical practice and improve their motivation to work. On the other hand, universities and clinical facilities need cooperation to help bridge the gap between theoretical and practical knowledge [25]. They can cooperate to hold case-based teaching to update students’ knowledge and skills, which can be effective in easing the transition process for nursing students.

#### 4.2.2. Night shift, first choice, and attitude toward nursing were associated with the dimension of overwhelming practicum workload

The participants in this study were exclusively from large tertiary A hospitals, characterized by a high volume of admitted patients and a complexity of disease types [26], thereby resulting in a significant workload and heightened care difficulty for nursing students. According to statistics, each nursing student was required to work an average of 3.96 ± 1.64 evening shifts and 3.32 ± 1.33 night shifts per month. In actual clinical practice, the role of nursing students was either nursing assistants or they did not seem to have a presence [25]. Because they were mostly occupied with tedious basic nursing tasks that tended to generate boredom, in the long run, their work motivation decreased and they experienced higher levels of transition shock. However, nursing students who choose nursing as their first choice and have a higher level of enjoyment of nursing have lower levels of transition shock compared to their peers due to their more positive attribution skills, willingness to take responsibility and adapt or ameliorate dilemmas when dealing with an overwhelming nursing workload [27]. It is recommended that nursing managers rationalize nursing students’ practicum schedules, humanize shift arrangements to balance the workload on each shift, and increase opportunities for nursing students to perform invasive operations.

#### 4.2.3. Attitudes toward nursing and first choice were associated with the dimension of loss of social support

The supply and demand in the nursing industry have remained unbalanced in recent years [28], yet advancements in medical and health services have led to improved social benefits and a greater familiarity with nursing. On this basis, nursing students join the nursing profession and obtain increasing social and family support. Moreover, students who choose the nursing profession as their first choice possess more confidence and initiative, which increases their internal motivation [29]. However, some students have less contact with their family, which leads to the loss of social support because of enormous stress and exhaustion [30]. It is recommended that internship mentors pay more attention to the psychological condition of nursing students, provide appropriate support, and create a good atmosphere in the department so that nursing students can integrate as soon as possible [31].

#### 4.2.4. Attitudes toward nursing, future plans, education, school scale, and first choice were associated with the dimension of shrinking interpersonal relationships

The influencing factors that were positively correlated with shrinking interpersonal relationships were attitude toward nursing, postgraduate development plan, and first choice, while the factors that were negatively correlated were educational background and scale of the school. Interpersonal relationships are related to the willingness of nursing students and their personal characteristics, and relationships are stronger when nursing students are willing to get along with others and support each other. However, the higher the level of education and the higher the level of the school scale, the greater the expectations of the people around them and the greater the corresponding pressure, which can lead to a lower level of interpersonal relationships [32].

#### 4.2.5. Attitudes toward nursing, future plans, monthly household income, and first choice were associated with the dimension of ambiguity in professional nursing values

Nursing students mainly play a student role before their internship. However, the values that nursing students develop in school are different from the clinical reality when they enter the hospital environment, which leads to a blurring of the role of nurses, disillusionment with the profession, and the experience of transition shock, resulting in thoughts of changing careers or confusion about future development plans. In a study by Kim and Song [33], nursing students said they thought about the future when considering themselves in relation to the newly graduated nurses they met in clinical practice. The participants felt disappointed that they were not treated as professionals in the actual work of new nurses and felt confused about their values [15]. Therefore, nursing students must fully think about the nursing profession. Directors of nursing and head nurses should also explore optimal clinical teaching methods, clinical teaching models, and teaching facilities to significantly reduce ambiguity in professional nursing values for nursing students.

#### 4.2.6. Student leader experience, attitude toward nursing, future plan, and first choice were associated with the dimension of incongruity between clinical practicum and personal life

The most relevant factor for this dimension was the presence of student leader experience, which was positively correlated. It has been shown that practice nurses with student leader experience have great transferability and can quickly adapt to clinical practice. This experience can provide the ability to express oneself verbally, organize and coordinate, and resolve emergencies. During clinical practice, schools, teachers, and peers place higher expectations on student leaders, which makes student leaders demand more of themselves in every aspect. This dimension is also influenced by the first choice, the attitude toward the nursing profession, and the intention to develop after graduation, all of which depend on the ability to coordinate life and practice. It is suggested that nursing students should receive relevant experience sharing so that they can understand the relationship between life and internship and learn to prioritize. At the same time, some studies suggest that nursing administrators should establish a robust training management mechanism. This involves providing nursing students with leadership experience with more opportunities to showcase their strengths, reasonably arranging the ratio of these students to others in internship groupings and on-call work, and encouraging student leaders to exemplify leadership and foster a supportive environment during internships [31].

### 4.3. Attitude toward the nursing profession had an independent influence on nursing students’ transition shock

The results of this study showed that attitude toward the nursing profession had an independent influence on nursing students’ transition shock. The more nursing interns enjoyed nursing, the lower their level of transition shock and vice versa, with a significant correlation. According to self-concordance theory, individuals’ behavior is guided by their decisions, which are bound by internal characteristics and the external environment. When the external environment is in line with the internal characteristics of individuals, individuals will see work as a kind of self-expression, which leads to a positive work attitude [34]. Our findings indicate that nursing undergraduates who possess a positive attitude towards their career aspirations and a sense of congruence between these aspirations and their academic experiences report lower levels of transition shock. What’s more, transition shock theory, which examines the process of adaptation during periods of change, provides a complementary perspective. The theory highlights how positive attitudes towards the nursing profession—such as enthusiasm for the field and commitment to professional development—affect individuals’ experience during the transition period. Our study finding also aligns with Transition Theory’s emphasis on the importance of positive attitudes and supportive systems in facilitating successful adaptation during transitional phases. Therefore, the social environment greatly affects nursing students’ attitudes toward the nursing profession. University education should help students build beliefs, while the clinical learning environment connects university study and clinical placements. A study showed that an environment that positively influences the choice of a future career allows nursing students to adapt quickly to the clinical pace [35]. Therefore, it is necessary to pay more attention to improving the education strategy and the practice plan and to focus more on two-way feedback.

### 4.4. Limitations

First, this study used a convenience sampling method, which may have led to large discrepancies and potential response bias, potentially affecting the generalizability of the findings. To address this limitation, future research could utilize random sampling techniques to reduce bias and enhance the external validity of the results. Additionally, replication studies in diverse settings are needed to validate and extend these findings. Second, the cross-sectional design of the study could only provide a temporal snapshot of transition shocks at a given point in time. To capture the dynamic nature of transition shock, a longitudinal study design could be employed to track changes over time. Third, the study used a single research method, which was limited by many objective factors. Moreover, no intervention was implemented in this study. A qualitative study or longitudinal survey could be conducted subsequently. Fourth, the participants were all from tertiary A hospitals in Changsha, so the findings were limited to one region. It is recommended that researchers conduct a multi-regional, national survey with a large sample size to develop normative data for the transition shock scale.

### 4.5 Implications for nursing & health policy

According to the results of the study, improving nursing students’ enjoyment of nursing may be meaningful for managing their transition shock. Therefore, nursing managers ought to strengthen the publicity of the nursing profession, so that more people can correctly understand the nursing industry, and improve the social recognition and authority of nursing. At the same time, more career development opportunities such as participating in academic conferences and scientific research projects should be provided, so as to give nursing students a broader space for growth. Nursing administrators should establish a sound training management mechanism, appropriately balancing the ratio of nursing students with leadership experience to others in internship groupings and on-call assignments, while fostering an environment where student leaders can exemplify leadership and support their peers during clinical practice. Taking the initiative to encourage students to relax, create a harmonious and pleasant atmosphere as well as focusing on the development of cooperation skills will also beneficial to their interpersonal relationships. Worth noting is that our findings could inform healthcare policy by emphasizing the importance of supportive frameworks for managing transitions, potentially guiding policy development to better support nursing professionals throughout their careers.

## 5. Conclusions

The current study demonstrated that Chinese nursing students’ transition shock level was close to the median on the transition shock scale. Nursing students’ attitudes toward nursing are a significant positive predictor of transition shock. Additionally, the number of night shifts, choosing nursing as the first choice, being class leaders, education level, future plans, school scale, and monthly household income contribute to different dimensions of transition shock. Our findings suggest that improving nursing students’ enjoyment of nursing may be meaningful for managing their transition shock. Clinical managers are recommended to implement structured onboarding, mentorship programs, regular feedback, and stress management resources to better support nursing students, improve their attitudes, and facilitate a successful transition into clinical practice. Additionally, nursing managers should provide physical, psychological, and social support to nursing students to help them successfully complete their role transition. What’s more, future research could explore the effects of specific interventions aimed at improving nursing students’ attitudes on alleviating transition shock.

## Supporting information

S1 Table The assignment of enumeration data. (DOC)

S2 Table Independent variable assignment. (DOC)

S1 File (XLSX)
