# Supplementary material for: Status and associations of transition shock among nursing students during clinical practice: A cross-sectional study
Source: PLoS One. 2025 Feb 4;20(2):e0313524. doi: 10.1371/journal.pone.0313524 (PMC11793765; doi:10.1371/journal.pone.0313524)
Supplement: S1 Table — (DOC) [file pone.0313524.s001.doc]

**Supplementary Information**

Table S1 The Assignment of Enumeration Data

| Enumeration data | Assignment |
| --- | --- |
| Attitude toward Nursing | “Strongly Like” =1; “Like”=2； “Average” =3；“Dislike” =4；“Strongly Dislike” =5 |
| First Choice | “Nursing” =1, “Others” =2 |
| Future Plan | “Clinical Nursing” =1; “Advanced Study” =2; “Nursing Education” =3; “Profession Change” =4; “Have not Decided” =5; “Others” =6 |
| Education | “Undergraduate” =1; “Junior College” =2; “Technical Secondary School” =3 |
| School Scale | “Key University” =1; “First Batch of University” =2; “Second Batch of University” =3; “Junior College” =4 |
| Monthly Household Income | “＜2500” =1; “2500-5000” =2; “5001-7500” =3; “＞ 7500”=4 |
| Student Leader Experience | “Yes” =1; “No” =2 |
