# Supplementary material for: Status and associations of transition shock among nursing students during clinical practice: A cross-sectional study
Source: PLoS One. 2025 Feb 4;20(2):e0313524. doi: 10.1371/journal.pone.0313524 (PMC11793765; doi:10.1371/journal.pone.0313524)
Supplement: S2 Table — (DOC) [file pone.0313524.s002.doc]

**Supplementary Information**

Table S2. Independent variable assignment (*N* = 564).

| Variables | Assignment |
| --- | --- |
| Monthly Household Income | Set dummy variable with “< 2500” as reference  Dummy variable  X1: “2500-5000” = 1; “< 2500” “5001-7500” “> 7500” = 0  Dummy variable  X2: “5001-7500” = 1; “< 2500” “2500-5000” “> 7500” = 0  Dummy variable  X3: “> 7500” = 1; “< 2500 ” “2500-5000” “5001-7500” = 0 |
| Present Intern Department | Set dummy variable with “Others” as reference  Dummy variable X1: “Internal Medicine” = 1; “Surgery” “Gynecology & Obstetrics” “Pediatrics” “Emergency & ICU” “Psychology” “Operating Room” “Others” = 0  Dummy variable X2: “Surgery” = 1; “Internal Medicine” “Gynecology & Obstetrics” “Pediatrics” “Emergency & ICU” “Psychology” “Operating Room” “Others” = 0  Dummy variable X3: “Gynecology & Obstetrics” = 1; “Internal Medicine” “Surgery” “Pediatrics” “Emergency & ICU” “Psychology” “Operating Room” “Others” = 0  Dummy variable X4: “Pediatrics” = 1; “Internal Medicine” “Surgery” “Gynecology & Obstetrics” “Emergency & ICU” “Psychology” “Operating Room” “Others” = 0  Dummy variable X5: “Emergency & ICU” = 1; “Internal Medicine” “Surgery” “Gynecology & Obstetrics” “Pediatrics” “Psychology” “Operating Room” “Others” = 0  Dummy variable X6: “Psychology” = 1; “Internal Medicine” “Surgery” “Gynecology & Obstetrics” “Pediatrics” “Emergency & ICU” “Operating Room” “Others” = 0  Dummy variable X7: “Operating Room” = 1; “Internal Medicine” “Surgery” “Gynecology & Obstetrics” “Pediatrics” “Emergency & ICU” “Psychology” “Others” = 0 |
| Education Background | Set dummy variable with “Undergraduate” as reference  Dummy variable X1: “Junior College” = 1; “Undergraduate” “Technical Secondary School” = 0  Dummy variable X2: “Technical Secondary School” = 1; “Undergraduate” “Junior College” = 0 |
| School Scale | Set dummy variable with “Key University” as reference  Dummy variable X1: “First Batch of University” ; “Key University” “Second Batch of University ” “Junior College” = 0  Dummy variable X2: “Second Batch of University” ; “Key University” “First Batch of University” “Junior College” = 0  Dummy variable X3: “Junior College” ; “Key University” “First Batch of University” “Second Batch of University” = 0 |
| Attitude toward Nursing | Set dummy variable with “Average” as reference  Dummy variable X1: “Strongly Like” = 1; “Like” “Average” “Dislike” “Strongly Dislike” = 0  Dummy variable X2: “Like” = 1; “Strongly Like” “Average” “Dislike” “Strongly Dislike” = 0  Dummy variable X3: “Dislike” = 1; “Strongly Like” “Like” “Average” “Strongly Dislike” =0  Dummy variable X4: “Strongly Dislike” = 1; “Strongly Like” “Like” “Average” “Dislike” = 0 |
| Future Plan | Set dummy variable with “Others” as reference  Dummy variable X1: “Clinical Nursing” = 1; “Advanced Study” “Nursing Education” “Profession Change” “Have not Decided” “Others” = 0  Dummy variable X2: “Advanced Study” = 1; “Clinical Nursing” “Nursing Education” “Profession Change” “Have not Decided” “Others” = 0  Dummy variable X3: “Nursing Education” = 1; “Clinical Nursing” “Advanced Study” “Profession Change” “Have not Decided” “Others” = 0  Dummy variable X4: “Profession Change” = 1; “Clinical Nursing” “Advanced Study” “Nursing Education” “Have not Decided” “Others” = 0  Dummy variable X5: “Have not Decided” = 1; “Clinical Nursing” “Advanced Study” “Nursing Education” “Profession Change” “Others” = 0 |
